# Food and nutrition knowledge, attitudes and practices among children in public primary school with canteens in southern Benin: a case study

**DOI:** 10.1186/s40795-024-00857-7

**Published:** 2024-03-04

**Authors:** Faïck Bello, Elie Koukou, Sam Bodjrenou, Céline Termote, Paulin Azokpota, Waliou Amoussa Hounkpatin

**Affiliations:** 1 School of Nutrition and Food Technology and Sciences, Faculty of Agricultural Sciences, University of Abomey-Calavi, Calavi, Benin; 2 Alliance of Bioversity International and CIAT, Abomey-Calavi, Calavi, Benin; 3 Alliance of Bioversity International and CIAT, Duduville Campus, Nairobi, Kenya

**Keywords:** School feeding, Nutrition education program, Public primary school, Benin

## Abstract

**Background:**

Food and nutrition notion among schoolchildren in Benin is still less documented. Few studies have examined the determinants of food and nutrition among schoolchildren while others have attempted to link knowledge, attitudes and practices to diet. The present study aims to evaluate food and nutrition knowledge, attitudes and practices among schoolchildren enrolled in public primary schools canteens in Cotonou and its surroundings.

**Methods:**

A diagnostic study was conducted in twelve public primary schools with canteens. A structured interview questionnaire was used to collect data from 861 schoolchildren aged 7 to 14 years. Three scores were used to assess the level of knowledge, attitude and practice. The overall score was the total of correct responses. The maximum score for all the three parameters was 15, 6 and 4 respectively for knowledge, attitude and practices. Data were analyzed using STATA 16. Logistic regression was performed to identify the relationship between food and nutrition practices and knowledge and attitude. Pearson goodness of fit test was performed to verify the adequacy of the model. A P-value of less than 0.05 (*P* < 0.05) was considered significant at 95% confidence interval.

**Results:**

schoolchildren’s nutrition knowledge was low (mean score 2.52 ± 1.33) while attitude and practices were acceptable (mean score 4.08 ± 1.39 and 2.84 ± 0.77). Only 18.2% of schoolchildren knew the different food groups and 3.4% knew that they should eat at least five fruits and vegetables a day. Most of the schoolchildren (93.6%) were favorable to eat at least five fruits and vegetables and 86.8% were willing to eat more than 3 times a day. Among all practices, snacking between meals and eating breakfast were poorly observed by the schoolchildren. Nutrition knowledge was associated with practices observed among schoolchildren but not with attitudes. However, a significant positive association was observed (*p* < 0.05) between attitudes and practices.

**Conclusion:**

Knowledge on food and nutrition among schoolchildren from public primary schools with canteen was low. This study suggests implementation of nutritional education to improve schoolchildren’s knowledge and attitudes towards healthy diets and nutrition.

## Background

Adequate and healthy nutrition is essential to reach full potential at every stage of growth and development but most studies focused on vulnerable groups often target infants, young children and women. However, malnutrition is also prevalent among older children or adolescents with serious consequences for both their health and academic performance. Statistics show that more than 372 million school-aged children around the world suffer from micronutrient deficiencies; almost 100 million of them reside in sub-Saharan Africa [1]. The most common micronutrient deficiencies are iron, vitamin A, iodine and zinc. Among schoolchildren, these micronutrient deficiencies could cause a lack of productivity and academic performance, often leading to dropping out of school. Another consequence could be a weakened immune system which can lead to an increase in infectious diseases or even extreme fatigue [2]. In addition, micronutrient deficiencies could be associated with delayed maturation, poor muscle strength leading to constraints in capacity for physical work, and reduced bone density later in life [3]. The period, considered also as a growth period with increased nutritional needs, deserves special attention as well [4, 5]. Thus the 7,000 days after a child’s second birthday provide ongoing opportunities for children to reach their full potential [6, 7]. Good food and nutrition practices are therefore essential for the healthy development of young people [6]. Moreover, the adoption of healthy eating practices in childhood favors not only optimal growth but also the development of good eating habits in adolescence and adulthood [8].

To effectively contribute to addressing the various types of malnutrition, children need to understand the role of healthy foods and how they affect their health and nutritional status. Studying the threesome knowledge-attitude-practices could be an effective model for improving food intake and nutrition [9]. This model suggests that knowledge is a prerequisite for practice change and as knowledge increases, attitudes begin to change and, over time, practices could change [9]. Nutritional interventions such as food and nutrition awareness-raising programs targeting schoolchildren are more likely to have positive long-term effects such as improved nutrition practices, reduced malnutrition problems and nutrition-related chronic diseases [10, 11]. Literature shows that nutrition education programs can significantly increase children’s nutritional knowledge and improve their eating practices [8, 12, 13].

In Benin, in 2018 nearly 10% of adolescents were stunted, 5.5% were wasted, and anemia affected one-third of the school population, i.e. 34% [14]. This situation deteriorated during the COVID-110 pandemic with increased food insecurity levels. From 2019 to 2022, the number of undernourished people and the number of people affected by malnutrition increased [15, 16]. How children perceive the concepts of healthy diets and nutrition is still unexplored. To our knowledge, few studies addressed the issue by trying to highlight the knowledge-attitude-practices model of food intake and nutrition among schoolchildren. The present study therefore, aims to evaluate firstly, the level of knowledge, attitude and practices of schoolchildren with regard to food intake and nutrition, and secondly, to explore the factors associated with the practices of schoolchildren in public primary schools canteen in urban (Cotonou) and peri-urban area (Abomey-Calavi and Sèmè-Kpodji) municipalities. These three areas are experiencing rapid urbanization and transition in dietary practices; while vegetable gardening is often practiced in urban and peri-urban areas in Benin. The public primary schools have been purposively selected because of the low socioeconomic status of the Schoolchildren’s households, the high prevalence of malnourished schoolchildren [14, 17, 18] and existence of school feeding programs through canteens. This program called (Programme National d’Alimentation Scolaire Intégrée PNASI) is funded by the government and currently implemented by the WFP. This program sets up a canteen in each beneficiary school and offers a meal to each student. To date, not all public primary schools are enrolled in the program (80% coverage rate). The present study focuses on the students who are enrolled in the public primary schools with canteen in three communes of southern Benin.

## Methods

### Study design and sampling

The present study is an observational descriptive cross-sectional study. The three municipalities, Cotonou, Abomey-Calavi and Sèmè-Kpodji (CAS) were purposively selected.

Charan and Biwas [19] formula was used to calculate the sample size at 5% level of significance and 80% power.

***n = 2(Zα/2 + Zβ)/δ***^***2***^ where ***α*** = 5%; ***β =*** 20%; and **δ**: detectable difference **=** 6 g/L.

The design effect was determined by the formula ***DE = 1+(K-1)∗ICC*** where (K = 75) represents the number of schoolchildren per cluster and ICC the Inter-Cluster Correlation fixed at 0.05 [20]. A sample size of 861 schoolchildren aged 7 to 14 years old was calculated.

The number of schools was then obtained by dividing the number of schoolchildren to be surveyed by the number of schoolchildren per cluster, i.e. 11.48 schools, hence the choice of 12 Public Primary Schools (PPS). We randomly selected these 12 PPSbased on the sampling frame from the list of all PPS benefiting from the National Integrated School Feeding Program (PNASI) implemented by the World Food Program and Benin Government in these three municipalities. Between the 58 PPS benefiting from canteens in CAS, we proceeded to a simple random selection of 12 PPS with canteens at the rate of four per municipality (Table 1). In each school, 75 schoolchildren in third, fourth and fifth grades were selected due to the reason that we did not want to disturb schoolchildren who were in exam class or those who were too young (under 7 years) to participate or answer questions.

**Table 1:** Public Primary Schools per municipalities

Municipalities	Cotonou	Abomey-Calavi	Sèmè-Kpodji
Public Primary Schools	Toyoymé, Yagbé A, Yagbé B, Lomnava	Dassekommey, Ouega-Tokpa, Ahossou-Gbèta Ahouato	Yagbé, Djeffa Plage, Kpakpakame Gbakpodji

### Data collection

Sociodemographique data (area of residence, socioeconomic level, father’s occupation, instruction level, mother’s age, sex of the household, schoolchildren age, grade) and data related to food and nutrition knowledge, attitude and practices were collected from December 2021 to February 2022. A structured questionnaire, based on FAO’s KAP questionnaire [21], was administered to the schoolchildren. The knowledge parameters considered were related to dietary diversity (different food groups), the role of vitamins and minerals, the definition of a healthy diet, the definition of malnutrition, the importance of fruit and vegetable consumption, recommended meal frequency and the consequences of malnutrition. Schoolchildren’s attitudes assessed were related to fruit and vegetable consumption, different food groups eating, having breakfast, having snacks, buying healthy foods or not, and recommended daily meal frequency. Practices were measured through the daily frequency of fruit and vegetable consumption, having breakfast, having snacks and daily frequency of food consumption. To minimize confounders, schoolchildren were randomly selected based on the list obtain from school authorities.

Data were collected electronically by enumerators after training and pre-testing using the World Bank *Survey Solutions* version 23.06. The enumerators were those who had “Fon” or “Goun” as their mother tongue or were fluent in these languages, which are the local languages spoken in the selected communities, and had at least bachelor’s degree in nutrition, sociology or agronomy.

### Data processing and statistical analysis

For each parameter of knowledge, attitudes and practices, correct/desired responses were coded ‘1’ and incorrect ones were coded ‘0’. For each aspect (knowledge, attitudes or practices), a score corresponding to the number of correct/desired responses was calculated [17]. For the interpretation of these scores, the thresholds used by Kigaru and colleagues were considered. The maximum scores for knowledge, attitudes and practices were 15, 6, and 4 respectively; scores ≤ 4, 5–10 and > 10 were categorized as low, moderate and high knowledge respectively; a score > 3 was considered as a positive attitude and a score > 2 was considered as good practices [17]. To access the factors associated to feeding practices the varaiable was transformed to a binary variable. Feeding practices score under the median was codified “0” for unadequate feeding practices and “1” for good feeding practices when the feeding practices were above the median score.

Data were analyzed using STATA 16. Bivariate analysis was used to assess the association between categorical variables using the Chi-square test. The student t-test was used for mean comparison. Logistic regression was performed to identify the relationship between food and nutrition practices and knowledge and attitude. Pearson goodness of fit test was performed to verify the adequacy of the model. A P-value of less than 0.05 (*P* < 0.05) was considered significant at 95% confidence interval.

## Results

### Demographic characteristics of schoolchildren

The age of schoolchildren ranged 7–14 years with a majority (21.25%) being 9 years old. The mean age was 9.47 ± 1.54 years. Girls represented 50.49% of the study sample. The average household size was 6.60 ± 2.45. Most of the households (86.5%) were headed by men. The average age of household heads was 43.6 ± 8.0 years old. (Table 2).

**Table 2:** Demographic characteristics of the schoolchildren

Variables	Characteristics	*n*	Percent (%)	Mean/SD
Gender	Boys	427	49.59	
	Girls	434	50.41	
Children Age (years)		861		9.47 ± 1.54
Household Size		861		4.36 ± 8.0
Household head	Fathers	743	86.50	
	Mothers	116	13.50	

### Food and Nutrition knowledge

**Fig. 1:**
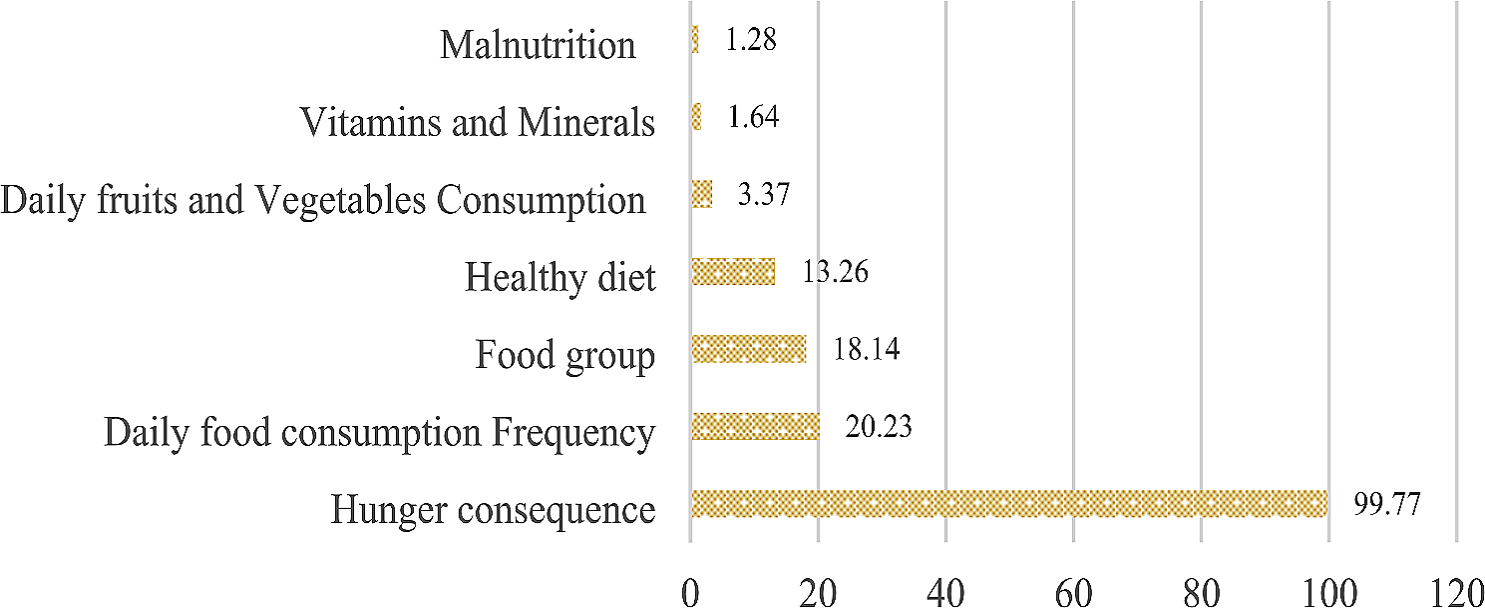
Food and nutrition knowledge among schoolchildren

The mean knowledge score was 2.52 ± 1.33 out of 15. The knowledge level was low for the majority of schoolchildren (91.17%) while 8.83% had moderate knowledge (Fig. 1). No schoolchildren had a high level of food and Nutrition knowledge. Indeed, less than one out five schoolchildren knew about the different food groups (18.14%) and the consumption of a healthy diet (13.26%). Barely a fifth of schoolchildren had good knowledge of the recommended daily meal frequency (20.23%). Less than 5% of schoolchildren had good knowledge of daily fruit and vegetable consumption, vitamins and minerals, and malnutrition. However, almost all schoolchildren succeeded in giving examples of the consequences of malnutrition.

### Food and Nutrition attitude

**Fig. 2:**
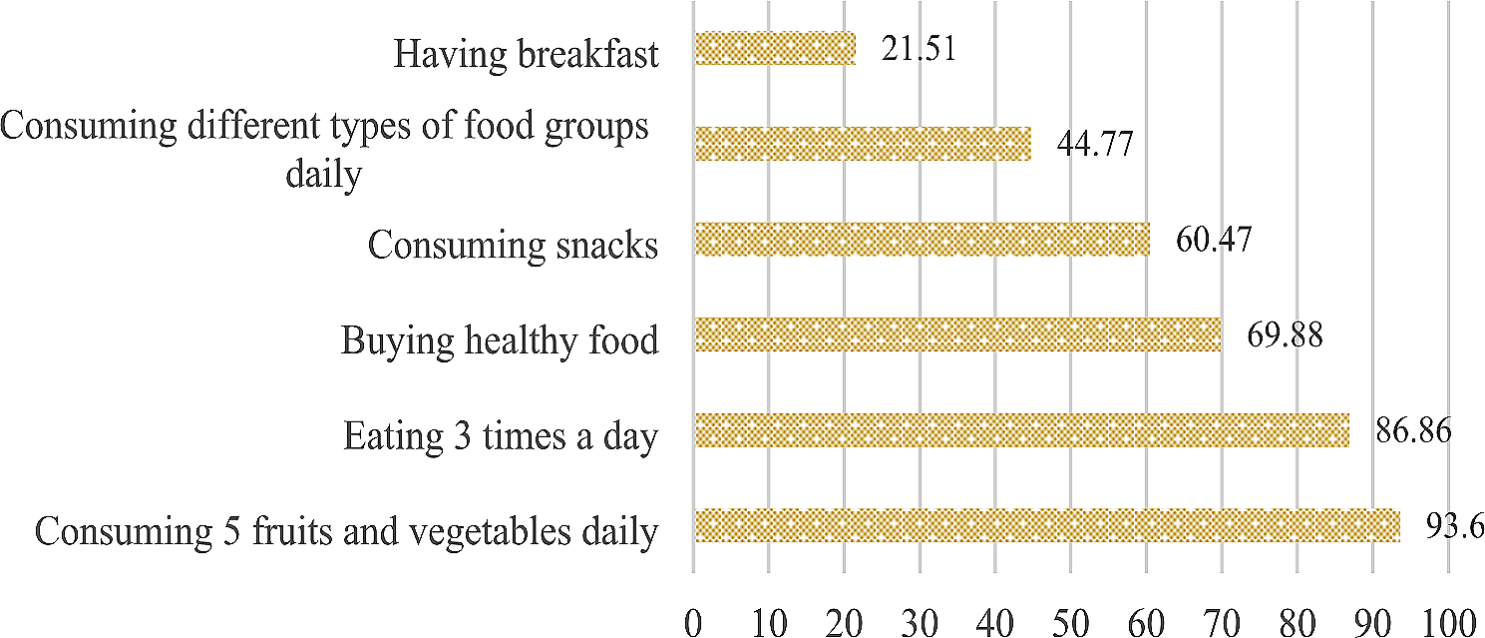
Food and nutrition attitude among schoolchildren

The average attitude score was 4.08 ± 1.39 out of six. Attitudes towards food intake and nutrition were positive for 71.66% of schoolchildren. Out of all observations (Fig. 2), schoolchildren had clear positive attitudes towards two practices. Most of the surveyed schoolchildren (93.6%) agreed on the fact that eating different types of fruits and vegetables daily is good, followed by the number of times they must eat within a day (86.86%).

#### Feeding practices

**Fig. 3:**
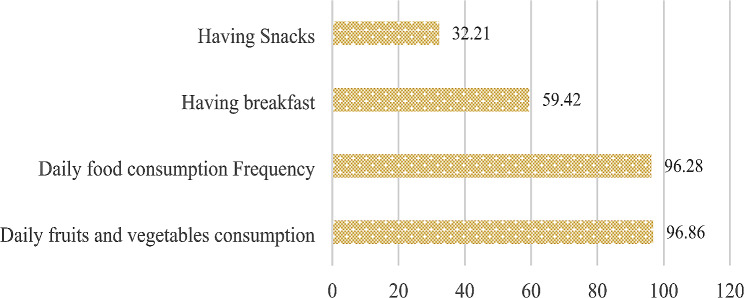
Feeding Practices among schoolchildren

The average score observed among the schoolchildren was 2.84 ± 0.77 with a minimum of zero and a maximum of four. 68% of the schoolchildren had desirable feeding practices. In terms of observed practices, eating at least one fruit and vegetable daily and eating three meals daily were parameters with the highest percentages. None of the surveyed schoolchildren reported consuming a minimum of five fruits and vegetables in the 24-hour recall period. Among all the schoolchildren, just over half (59.41%) reported having breakfast before going to school and one-third (32.21%) had a snack during the day (Fig. 3).

### Factors associated with adequate feeding practices

Schoolchildren’s desired feeding practices improved with the age of mothers (OR = 1.03; *P* = 0.010) and were also associated with the area of residence (OR = 1.92; *P* = 0.000). Schoolchildren living in urban areas were almost twice more likely to have good practices than schoolchildren living in peri-urban areas (Table 3).

Food and nutrition practices were significantly associated with knowledge and attitude. Schoolchildren with positive attitudes were 2.19 times more likely to have adequate feeding practices than those with a negative attitude. The association between practices and knowledge was negative; schoolchildren with good knowledge were 0.48 times less likely to have adequate feeding practices (Table 3).

**Table 3:** Factors associated with adequate feeding practices

Feeding practices	Odds Ratio	*P*-Value	Confidence Interval
Attitudes (inadequate)	-	-	-
Attitudes (good)	2.19	0.000	1.55–3.10
Knowledge (low)	-	-	-
Knowledge (middle)	0.48	0.006	0.29–0.81
Knowledge (high)	-	-	-
Area of residence (peri-urban)	-	-	-
Area of residence (urban)	1.92	0.000	1.34–2.75
Schoolchildren’s age	0.84	0.001	0.75–0.93
Mother’s age	1.03	0.010	1.01–1.06

Pearson goodness of fit (chi2) = 0.59

## Discussion

The present study aimed to evaluate schoolchildren’s food knowledge, attitudes and practices and to highlight the determinants of food and nutrition practices.

Overall, a majority of 91% of schoolchildren have low food and nutrition knowledge, while attitude ($\sim$ 72%) and practices ($\sim$ 68%) were much better. This situation might be explained by the lack of food and nutrition lessons taught at school, specially at grade 3 and 4, and also by the fact that teachers and parents particularly mothers who are responsible for children’s education are not knowledgeable enough about food and nutrition aspects. However some few food and nutrition aspect are teach in primary school but specially with schoolchildren in fifth and sixth grade. Similar trends were observed in the studies conducted in Kenya and South Africa [9, 18, 22]. A pre-post controlled design conducted in Accra, Ghana [23] shows as other studies [24, 25] that teachers’ or mothers’ knowledge or their implication in the education of schoolchildren could have a positive impact on schoolchildren’s knowledge. In contrast, a study conducted in Nairobi [17] found that most schoolchildren’s nutrition knowledge level was moderate and this finding was due to the fact that health lessons were offered in school. In Teheran, a similar finding was attributed to the fact that schoolchildren obtained their nutritional knowledge from their parents [26]. Our study, however, did not look at parents’ and teachers’ food and nutrition knowledge; but these findings highlight the important role of parents and schools in the nutritional education of children.

Schoolchildren’s attitude in terms of food and nutrition was quite good. Most of them feel comfortable with consuming more fruits and vegetables or eating more than three times daily. They were also in favor of eating different food groups. This situation could be explained firstly by the fact that there are probably no cultural beliefs or taboos that obliged them to not consume a certain type of food group and secondly, the school food environment offers different types of food that are available but not necessarily affordable. The same trend was observed in a study conducted in Kajiado district in Kenya [27]. However, a contrary trend was observed in studies conducted in other countries [9, 18] where schoolchildren’s attitudes towards desirable food and nutrition practices were negative. This situation was explained by the food groups sold in their food environment and the lack of knowledge. Schoolchildren do not have the habit of making a lunch box; they rather carry money to school to buy snacks and sweet drinks.

The present study also finds that feeding practices regarding some aspects observed among schoolchildren were acceptable and significantly associated to knowledge and attitude. Most of them ate at least three times per day and usually had breakfast. This could be due to their food habits, the fact that they carry money to school and the meals offered in the canteen (rice + tomato sauce, maize dough + tomato or leafy vegetables sauce, granulated fermented cassava + sugar, macaroni, cowpeas, yellow peas, etc.). The same trend was observed in a study conducted by Lin et al. [28] in Taiwan. Our study shows that most schoolchildren ate one fruit or vegetable daily but none of them observed the World Health Organization (WHO) recommendation of five fruits and vegetables or 400 g of fruits and vegetables daily and few (32%) of them took snacks. The low fruit and vegetable consumption could be explained by the inadequate food habits, the lack of knowledge or the low availability of fruits and vegetables in their local food environment wich can be at school or home. Studies conducted in seven African countries [29] and five Southeast Asian countries [30] found the same trends where children’s consumption of fruit and vegetables was poor.

The present study reveals that knowledge and attitude influence schoolchildren’s practices. Schoolchildren with average knowledge of food and nutrition have 0.48 time less desirable practices than those having inadequate knowledge. This situation could be explained by the fact that the school food environment is not diversified [31]. Even though schoolchildren with average food and nutrition knowledge were in higher grades, they carried money to school and instead of buying healthy food they bought snacks and sweet drinks that were available. A study conducted in Teheran [26] found that even though schoolchildren had some level of knowledge on the effect of unhealthy diets on their health, they continued to consume unhealthy diets because their friends did. Other studies have shown nutritional knowledge as a factor that influences the individual’s food choices [22, 32].

Attitude among schoolchildren was positively associated with practices. Schoolchildren who had positive attitudes had 2.19 times better practices than others in terms of food and nutrition. The same trend was observed in a study conducted in Slovenia [33] where they found that a positive attitude to healthy dietary habits encourages children to follow recommendations taken as healthy eating habits, while a negative attitude to unhealthy eating habits can prevent unhealthy forms of nutrition practices. This implies that the more they have positive attitudes towards healthy diets, the better is their practices. Positive attitudes towards good nutrition play an important role in improving nutrition practices among school children.

The results of this study highlight the aspects on which we have to focus our efforts to contribute to the improvement of schoolchildren’s food intake and nutrition. The school food environment remains one of these aspects that deserves improvement. Although some schoolchildren have an average level of knowledge in food and nutrition, they do not hesitate to buy sweets and candies available in their school environment. It is therefore not enough to have a good knowledge of food and nutrition to have a good diet, you should also have access to healthy and nutritious food. By acting on the quality of the food supply in public primary schools and by working in closer collaboration with both school officials and vendors, we could, on one hand, reduce the availability of foods that are not very nutritious or ultra-processed food and on the other hand to increase the supply of desired and healthy foods in schools. Further studies must be carried out to better appreciate the role of the school food environment in all its components in school context.

Moreover, the positive correlation, highlighted in the present study, existing between the positive attitude and the recommended feeding practices, gives a glimpse of hope for improving food practices among schoolchildren. The establishment of a food and nutrition education program aimed at improving knowledge and attitude could be an effective approach to improve the food choices made by school children.

### Limitations

This study has some limitations. The first is linked to the fact that the study only concerned public primary schools with canteens and secondly it did not take into account the daily consumption of five fruits and vegetables but rather the daily consumption of at least one fruit or vegetable.

## Conclusion

Knowledge on food and nutrition among schoolchildren from public primary schools with canteen was low. Schoolchildren did not know much about the different types of food groups, the desired fruits and vegetables consumption, a healthy diet, or malnutrition, but they knew more about the consequences of hunger. Although schoolchildren’s attitudes were generally favorable to healthy diets and nutrition, they remain reluctant to eat breakfast and to increase their consumption of fruits and vegetables. Practices such as eating at least one fruit or vegetable a day or eating three times a day were observed as being good. However, eating breakfast and healthy snacks should be strongly encouraged among schoolchildren. Furthermore, significant associations were observed between knowledge, attitudes and practices. These findings suggest an implementation of nutritional education to improve schoolchildren’s knowledge and attitudes toward healthy diets and nutrition.

## Data Availability

The datasets used and analyzed during the current study are available with the corresponding author.

## Declarations

The present study was approved by the National Ethics and Health Research Committee (Authorization N° 53/MS/SGM/CNERS/SA, 2021) located in Akpakpa (Cotonou) which is one of our study areas and the Ministry of Nursery (Pre-school) and Primary Education (Authorization N^o^ 0031/MEMP/DC/DEP/SAS/SP, 2021). Moreover, each schoolchild had given his assent to participate and parents had provided aa written informed consent before starting the data collection.

## Abbreviations

CAS: Cotonou Abomey-Calavi Seme-kpodji
CNERS: National Ethics Committee for Health Research
DC: Director of Cabinet
DEP: Director of Primary Education
ICC: Inter Correlation Cluster
MEMP: Ministry of Nursery and Primary Education
MS: Health Ministry
OR: Odds Ratio
SA: Administrative Secretary
SAS: Special Administrative Secretary
SD: Standard Deviation
SGM: General Secretariat of the Ministry
SP: Particular Secretary
